# Polarization: A Key Difference between Man-made and Natural Electromagnetic Fields, in regard to Biological Activity

**DOI:** 10.1038/srep14914

**Published:** 2015-10-12

**Authors:** Dimitris J. Panagopoulos, Olle Johansson, George L. Carlo

**Affiliations:** 1 National Center for Scientific Research “Demokritos”, Athens, Greece; 2 Department of Biology, University of Athens, Greece; 3 Radiation and Environmental Biophysics Research Centre, Greece; 4 Experimental Dermatology Unit, Department of Neuroscience, Karolinska Institute, Stockholm, Sweden; 5 The Science and Public Policy Institute, Institute for Healthful Adaptation, Washington, DC, USA

## Abstract

In the present study we analyze the role of polarization in the biological activity of Electromagnetic Fields (EMFs)/Electromagnetic Radiation (EMR). All types of man-made EMFs/EMR - in contrast to natural EMFs/EMR - are polarized. Polarized EMFs/EMR can have increased biological activity, due to: 1) Ability to produce constructive interference effects and amplify their intensities at many locations. 2) Ability to force all charged/polar molecules and especially free ions within and around all living cells to oscillate on parallel planes and in phase with the applied polarized field. Such ionic forced-oscillations exert additive electrostatic forces on the sensors of cell membrane electro-sensitive ion channels, resulting in their irregular gating and consequent disruption of the cell’s electrochemical balance. These features render man-made EMFs/EMR more bioactive than natural non-ionizing EMFs/EMR. This explains the increasing number of biological effects discovered during the past few decades to be induced by man-made EMFs, in contrast to natural EMFs in the terrestrial environment which have always been present throughout evolution, although human exposure to the latter ones is normally of significantly higher intensities/energy and longer durations. Thus, polarization seems to be a trigger that significantly increases the probability for the initiation of biological/health effects.

## Introduction

### Man-Made EMR is more Active biologically than Natural Non-Ionizing EMR

A large and increasing number of studies during the past few decades have indicated a variety of adverse biological effects to be triggered by exposure to man-made EMFs, especially of radio frequency (RF)/microwaves, and extremely low frequency (ELF). The recorded biological effects range from alterations in the synthesis rates and intracellular concentrations of different biomolecules, to DNA and protein damage, which may result in cell death, reproductive declines, or even cancer^1^,^2^,^3^,^4^,^5^,^6^,^7^. Under the weight of this evidence the International Agency for Research on Cancer (IARC) has classified both ELF magnetic fields and RF EMFs as possibly carcinogenic to humans^8^,^9^. The intensities of radiation and durations of exposure in all these studies were significantly smaller than those of corresponding exposures from natural EMFs in the terrestrial environment. Moreover, the field intensities applied in the studies were several orders of magnitude smaller than physiological fields in cell membranes, or fields generated by nerve and muscle excitations^10^,^11^.

Solar EMR intensity incident upon a human body ranges normally between 8 and 24 mW/cm^2^ (depending on season, atmospheric conditions, geographical location, etc) while corresponding intensity from a digital mobile phone handset upon a human head during “talk” emission is normally less than 0.2 mW/cm^2 ^ (Refs.  6,12,13). Similarly, terrestrial electric and magnetic fields, or infrared radiation from every human body at normal temperature, have significantly larger incident intensities and exposure durations on any human than most artificial EMF sources^14^,^15^,^16^. Why is then the first beneficial while the latter seem to be detrimental? In the present study we shall attempt to explain theoretically that the increased adverse biological action of man-made EMFs is due to the fact that they are polarized in contrast to the natural ones.

### Man-Made EMR is Polarized, while Natural EMR is not

A field/wave is called linearly polarized when it oscillates on a certain plane which is called the “polarization plane”. A combination of linearly polarized fields/waves can give circularly or elliptically polarized fields/waves.

Natural EMR/EMFs (cosmic microwaves, infrared, visible light, ultraviolet, gamma rays) and several forms of artificially triggered electromagnetic emissions (such as from light bulbs with thermal filaments, gas discharge lamps, x-rays, lasers, etc.) are not polarized. They are produced by large numbers of molecular, atomic, or nuclear transitions of random orientation and random phase difference between them (except for the lasers which are coherent). These are de-excitations of molecules, atoms, or atomic nuclei^17^. Each photon they consist of oscillates on a distinct random plane, and therefore it has a different polarization. Moreover the different photons are not produced simultaneously but they have random phase differences among them.

In contrast, man-made electromagnetic waves are produced by electromagnetic oscillation circuits (“Thomson” circuits), forcing free electrons to oscillate back and forth along a metal wire (electric circuit). Thus, they are not produced by excitations/de-excitations of molecules, atoms, or nuclei, and because the electronic oscillations take place in specific directions/orientations they are polarized (most usually linearly polarized). The plane of polarization is determined by the geometry of the circuit. [Lasers are coherent light emissions, not necessarily polarized, and condensed within a narrow beam with high intensity, but they may also be polarized]. Superposition of two fields of identical frequency and linear polarizations, equal amplitudes, and a phase difference 90° between them, or superposition of three such fields with a phase difference 120° between each two of them, and with specific geometrical arrangement, results in a circularly polarized field of the same frequency. The above combinations with unequal amplitudes results in elliptically polarized field of the same frequency^18^. Circularly and elliptically polarized 50–60 Hz electric and magnetic fields are formed around 3-phase electric power transmission lines. These fields are accused for an association with cancer^7^,^8^.

Oscillating polarized EMFs/EMR (in contrast to unpolarized) have the ability to induce coherent forced-oscillations on charged/polar molecules within a medium. In case that the medium is biological tissue, the result is that all charged molecules will be forced to oscillate in phase with the field and on planes parallel to its polarization^19^,^20^. Several oscillating electromagnetic fields of the same polarization - such as the fields from different antennas vertically oriented - may also produce constructive interference effects and thus, amplify at certain locations the local field intensity, and the amplitude of oscillation of any charged particle within the medium (and within living tissue). At such locations, living tissue becomes more susceptible to the initiation of biological effects^21^.

Only coherent polarized fields/waves of the same polarization and frequency are able to produce standing interference effects (fringes of maximum and minimum intensity)^22^. When the polarization is fixed (e.g. vertically oriented antennas) but there are differences in coherence and/or frequency between the sources, the interference effects are not standing at fixed locations, but change with time creating transient peaks at changing locations.

Natural light from two or more different sources does not produce interference effects, except under the specific conditions of the Young experiment, where the light from a single source passes through two identical slits which - in turn - become two identical-coherent secondary sources^18^,^23^.

Unpolarized electromagnetic radiation can become polarized when it passes through anisotropic media, as are certain crystals. In fluids (gases and liquids) the molecules are randomly oriented, and macroscopically are considered isotropic inducing no polarization in the electromagnetic waves transmitted through them. Unpolarized natural light can become partly polarized to a small average degree after diffraction on atmospheric molecules, or reflection on water, mirrors, metallic surfaces, etc.^18^. Thus, living creatures exposed to natural radiation since the beginning of life on Earth, although have been exposed to partially polarized light at a small average degree under certain circumstances^24^,^25^, have never been exposed to totally polarized radiation as is EMR/EMFs of modern human technology.

### Field Intensity versus Wave Intensity of electromagnetic waves

A plane harmonic electromagnetic wave in the vacuum or the air has electric and magnetic field intensity components, given by the equations:


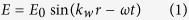



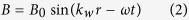


*r* is the distance from the source, *t* is the time, *ω* = *2πν* = *k*_*w*_ · *c*, is the circular frequency of the wave (*ν* the frequency), and *k*_*w*_(=*2π/λ*) is the wave number (*λ* the wavelength).

The velocity of the electromagnetic wave (and of any wave), is:


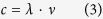


The wave intensity 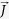 (“Poynting vector”), is:


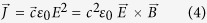


And the average value of its amplitude:


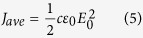


Thus, the wave intensity depends upon the square of the electric field intensity.

## Superposition of Electromagnetic Waves/Fields

### Superposition of Unpolarized EMR/EMFs

Consider two incoherent, unpolarized electromagnetic rays with electric components *E*_1_, *E*_2_, reaching a certain point P in space at a certain moment *t* in time. Let us assume for simplicity that the two waves are plane harmonic. The two vectors 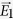, 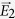 due to the different polarizations oscillate on different planes. Since the two waves are not polarized, their polarizations vary randomly with time. The total angle *ϕ* between the two vectors each moment is determined by the different polarizations, plus the different phases, and varies randomly in time.

The resultant electric field 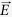 (electric component of the resultant electromagnetic wave) each moment at point P, is given by the equation:


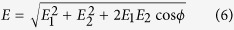


*E* varies with time due to the temporal variations of *E*_1_, *E*_2_, cos *ϕ*. But the average value of cos *ϕ* is zero:


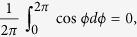


and the averages of *E*^2^, 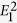, and 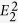 are 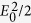, 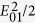 and 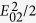 respectively (*E*_0_, *E*_01_, *E*_02_ the amplitudes of *E*, *E*_1_, *E*_2_).

The average resultant electric field is then:


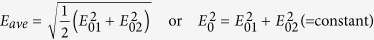


and (according to Eq. 5):


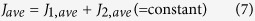


Even when the two component waves have the same frequency and phase, due to the randomly changing polarizations, the result is still the same.

Thus, the total time average wave intensity due to the superposition of two (or more) rays of random polarizations (natural EMR/EMFs) is the sum of the two individual average intensities, and it is constant at every point and - macroscopically - there is no local variation in the resultant intensity, i.e. no interference effects.

### Wave Intensity versus Field Intensity of Unpolarized EMR

Although the sum average wave intensity due to superposition of natural unpolarized waves is the sum of individual average intensities each one depending on the square amplitude of individual electric field (Eq. 7), the sum electric field from an infinite number of individual waves (as e.g. with natural light), is zero:


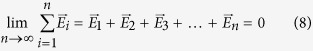


Let us explain this in more detail: Consider many photons of natural unpolarized light superposed on each other at a particular point in space. Let us assume for simplicity that these photons have equal amplitudes and are of the same frequency but have different polarizations meaning that their electric vectors have all possible orientations forming angles between each two of them from 0° to 360°. Since all possible orientations have equal probabilities, the superposition of a large number of such equal vectors applied on the same point in space will be the sum of vectors applied on the centre of a sphere with their ends equally distributed around the surface of the sphere. The sum of an infinite number of such vectors (all applied on the same point – centre of the sphere – and with their ends evenly distributed at all points of the sphere surface) tends to become zero.

In other words, at any given location, any moment, the sum electric field of a large number of incident photons of random polarization tends to be null, since the individual vectors are in all possible directions diminishing each other when superimposed (destructive interference of electric vectors). Similarly for the sum magnetic field:


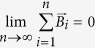


Thus, the result of superposition of a large number of incident natural waves is increased wave intensity, but negligible electric and magnetic fields approaching zero with infinite number of individual waves/photons. Since the electric forces on charged particles depend directly on electric and magnetic field intensities 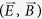, but not on the wave intensity 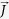, unpolarized EMFs/EMR cannot induce any net forced-oscillations on any charged particles (e.g. biological molecules). They may only induce heat, i.e. random oscillations in all possible directions due to momentary non-zero field intensities, but this does not result to any net electric or magnetic field, or to any net forced-oscillation of charged molecules.

### Superposition of Coherent Polarized Waves/Fields of the same polarization

When two or more waves/fields of the same polarization and frequency are in addition coherent, in other words, when their phase difference at the location of superposition is:


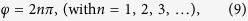


the result is constructive interference, meaning that the resultant wave has an amplitude (intensity) equal to the sum of amplitudes of the single waves that interfere at the particular location.

When two waves of same polarization have opposite phases at another location, in other words, when their phase difference is:


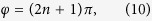


then the result of their superposition is destructive interference, i.e. a wave of the same polarization but with diminished intensity.

The electrical components of two such waves (plane harmonic waves of the same polarization and frequency) reaching a certain location after having run different distances *r*_1_, and *r*_2_ from their two coherent sources, are given by the equations:


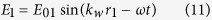



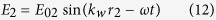


Again, the amplitude *E*_0_ of the resultant electric field 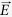 (electric component of the resultant electromagnetic wave), is:


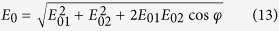


where 
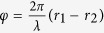
 depending in this case only upon the difference in the distances run by the two waves, and not upon polarization.

At any location where: *φ* = 2*nπ*, Eq. 13 gives:


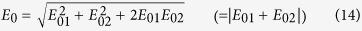


At these locations we have constructive interference.

At any location where: *φ* = (2*n* + 1)*π*, Eq. 13 gives:


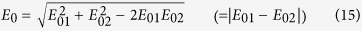


At these locations we have destructive interference.

The intensity of the resultant wave at any location is:


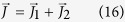


The amplitude of the resultant wave intensity will be, correspondingly:


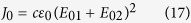


(at the locations of constructive interference), and


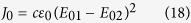


(at the locations of destructive interference).

Thus, at the locations of constructive interference, the electric field vectors of the two waves/fields are parallel and in the same direction, and both the resultant field and the resultant wave intensity are maximum (Eqs. 14 and 17).

For two identical sources (*E*_01_ = *E*_02_): *E*_0_ = 2*E*_01_ and 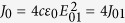


For *N* identical sources:


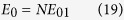


and:


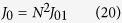


This is why series of parallel RF/microwave antennas are often used to produce high-intensity beams in certain directions^18^.

At the locations of destructive interference the electric field vectors of the two waves are anti-parallel, and thus, both the resultant field and the resultant wave intensity are minimum (Eqs. 15 and 18). For identical sources (*E*_01_ = *E*_02_): *E* = 0, *J* = 0.

Thus, for *N* number of polarized coherent electromagnetic sources of the same polarization, frequency, and different intensities, with electric components *E*_1_, *E*_2_, …, *E*_*N*_, it comes that at the locations of constructive interference, the resultant electric field is the sum electric field from all the individual sources (e.g. antennas):


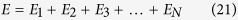


The bigger the number of coherent superimposed waves/fields (from the same or different sources), the higher and narrower the peaks^18^. That situation can create very sharp peaks of wave and field intensities at certain locations, not easily detectable by field meters, where any living organism may be exposed to peak electric and magnetic field intensities. Such locations of increased field/radiation intensity, also called “hot spots”, were recently detected within urban areas, due to wave/field superposition from mobile telephony base towers^21^. Any location along the midperpendicular to the distance *d* between two antennas is a location of constructive interference in the case of two identical antennas.

Thus, the difference between superposition of unpolarized and polarized electromagnetic waves/fields, is that while in the first case we have increased average wave intensity but zeroed net fields at any location, in the second case we have increased both wave intensity and fields at certain locations where constructive interference occurs. This difference is of crucial importance for understanding the differences in biological activity between natural and man-made EMFs/non-ionizing EMR.

## Induction of Forced-Oscillations in living tissue by Polarized EMFs

All critical biomolecules are either electrically charged or polar^11^. While natural unpolarised EMF/EMR at any intensity cannot induce any specific/coherent oscillation on these molecules, polarized man-made EMFs/EMR will induce a coherent forced-oscillation on every charged/polar molecule within biological tissue. This is fundamental to our understanding of the biological phenomena. This oscillation will be most evident on the free (mobile) ions which carry a net electric charge and exist in large concentrations in all types of cells or extracellular tissue determining practically all cellular/biological functions^11^. Although all molecules oscillate randomly with much higher velocities due to thermal motion, this has no biological effect other than increase in tissue temperature. But a coherent polarized oscillation of even millions of times smaller energy than average thermal molecular energy^26^ can initiate biological effects.

A forced-oscillation of mobile ions, induced by an external polarized EMF, can result in irregular gating of electrosensitive ion channels on the cell membranes. That was described in detail in Panagopoulos *et al*.^19^,^20^. According to this theory - the plausibility of which in actual biological conditions was verified by numerical test^27^ - the forced-oscillation of ions in the vicinity of the voltage-sensors of voltage-gated ion channels can exert forces on these sensors equal to or greater than the forces known to physiologically gate these channels. Irregular gating of these channels can potentially disrupt any cell’s electrochemical balance and function^11^, leading to a variety of biological/health effects including the most detrimental ones, such as DNA damage, cell death, or cancer^28^.

Most cation channels (Ca^+2^, K^+^, Na^+^, etc) on the membranes of all animal cells, are voltage-gated^11^. They interconvert between open and closed state, when the electrostatic force on the electric charges of their voltage sensors due to transmembrane voltage changes, transcends some critical value. The voltage sensors of these channels are four symmetrically arranged, transmembrane, positively charged helical domains, each one designated S4. Changes in the transmembrane potential on the order of 30 mV are normally required to gate electrosensitive channels^29^,^30^. Several ions may interact simultaneously each moment with an S4 domain from a distance on the order of 1 nm, since - except for the single ion that may be passing through the channel pore when the channel is opened - a few more ions are bound close to the pore of the channel at specific ion-binding sites (e.g. three in potassium channels)^31^. Details on the structure and function of cation electrosensitive channels can be found in^11^,^29^,^31^.

Consider e.g. four potassium ions at distances on the order of 1 nm from the channel-sensors (S4), and an externally applied oscillating EMF/EMR. The electric (and the magnetic) force on each ion due to any unpolarized field is zero (Eq. 8). In contrast, the force due to a polarized field with an electrical component *E*, is *F* = *Ezq*_*e*_. For a sinusoidal alternating field *Ε* = *Ε*_0_ sin *ωt*, the movement equation of a free ion of mass *m*_*i*_, is^19^,^20^:


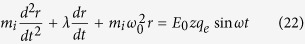


where *r* is the ion displacement due to the forced-oscillation, *z* is the ion’s valence (*z* = 1 for potassium ions), *q*_*e*_ = 1.6×10^−19^ C the elementary charge, *λ* the damping coefficient for the ion displacement (calculated to have a value within a channel 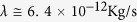), *ω*_0_ = 2*πν*_0_  (*ν*_0_  the ion’s oscillation self-frequency taken equal to the ion’s recorded spontaneous intracellular oscillation frequency on the order of 0.1 Hz), *ω* = 2*πν* (*ν* the frequency of the field/radiation), and *E*_0_ the amplitude of the field^19^,^20^.

The general solution of Eq. 22, is^19^,^20^:


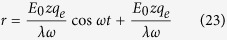


The term 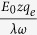 in the solution, represents a constant displacement, but has no effect on the oscillating term 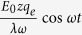. This constant displacement doubles the amplitude 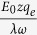 of the forced-oscillation at the moment when the field is applied or interrupted, or during its first and last periods, and the ion’s displacement will be twice the amplitude of the forced-oscillation. For pulsed fields (such as most fields of modern digital telecommunications) this will be taking place constantly with every repeated pulse. Thus, pulsed fields are - theoretically - twice more drastic than continuous/non-interrupted fields of the same other parameters, in agreement with several experimental data^1^,^32^.

The amplitude of the forced-oscillation (ignoring the constant term in Eq. 23), is:


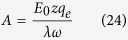


The force acting on the effective charge *q* of an S4 domain, via an oscillating single-valence free cation, is: 
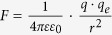
, (*r* is the distance of the free ion from the effective charge of S4). Each oscillating cation displaced by *dr*, induces a force on each S4 sensor:


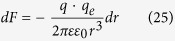


While in the case of a non-polarized applied field 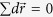, and 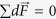, in the case of a polarized applied field, the sum force on the channel sensor from all four cations, is:


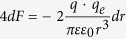


This is an even more crucial difference between polarized and unpolarized EMFs in regard to biological activity than the ability of interference.

The effective charge of each S4 domain is found to be: *q* = 1.7 *q*_*e*_^30^. The minimum force on this charge required normally to gate the channel - equal to the force generated by a change of 30 mV in the membrane potential^30^ - is calculated^19^ to be:


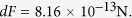


The displacement of one single-valence cation within the channel, necessary to exert this minimum force is calculated from Eq. 25 to be:


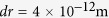


For 4 cations oscillating in phase and on parallel planes due to an external polarized field/radiation, the minimum displacement is decreased to: *dr* = 10^−12^ m.

Therefore, any external polarized oscillating EMF able to force free ions to oscillate with amplitude 
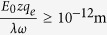
, is able to irregularly gate cation channels on cell membranes. For *z* = 1 (potassium ions), and substituting the values for *q*_*e*_, *λ* on the last condition, we get:


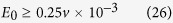


(*ν* in Hz, *Ε*_0_ in V/m)

For double-valence cations (*z* = 2) (e.g. Ca^+2^) the condition becomes,


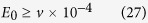


(*ν* in Hz, *Ε*_0_ in V/m)

[An in depth description of the briefly presented mechanism can be found in^19^,^20^.]

For electric power fields (*ν* = 50 Hz), Condition 27 becomes,


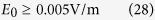


Thus, power frequency EMFs with intensities exceeding 5 mV/m are potentially able to disrupt cell function. For *N* number of EMF-sources of the same polarization (e.g. *N* number of parallel power lines) the last value is divided by *N* (according to Eq. 19) at the locations of constructive interference, and thus even more decreased. Such minimum power frequency field intensity values are abundant in urban daily environments, and even more close to high-voltage power transmission lines^7^.

For pulsed fields the second part of Condition 27 is divided by 2, and becomes:


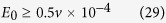


(*ν* in Hz, *Ε*_0_ in V/m).

For digital mobile telephony fields/radiation emitting ELF pulses with a pulse repetition frequency *ν* = 217 Hz (among other ELF frequencies they transmit)^33^, Condition 29 becomes:


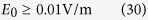


For the pulse repetition frequency of *ν* = 8.34 Hz (also included in mobile telephony signals)^33^,^34^, Condition 29 becomes:


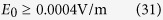


As is evident from the described mechanism, the field does not gate the channel by forces exerted directly on the channel sensors. It would take a field on the order of the transmembrane field (10^6^–10^7^ V/m) for that. It is the mediation of the oscillating free ions in close proximity to the S4 channel sensors that allows such weak fields to be able to exert the necessary forces to gate the channel.

Thus, ELF electric fields emitted by mobile phones and base stations stronger than 0.0004 V/m are also potentially able to disrupt the function of any living cell. This ELF intensity value is emitted by regular cell phones at distances up to a few meters and base stations at distances up to a few hundred meters^6^,^34^,^35^. For *N* number of mobile telephony antennas vertically oriented, the last value is divided by *N* (according to Eq. 19) at locations of constructive interference.

We do not distinguish between externally applied EMFs and internally induced ones within living tissue, especially in the case of ELF for the following reasons: 1. Living tissue is not metal to shield from electric fields and certainly is not ferromagnetic metal (Fe, Co, Ni) to shield from magnetic fields. Moreover, it is known that especially ELF fields cannot be easily shielded even by Faraday cages and in order to significantly minimize them it is recommended to totally enclose them in closed metal boxes^6^. Thus, ELF electric fields penetrate living tissue with certain degree of attenuation, and magnetic fields penetrate with zero attenuation. 2. Even in case that the ELF fields are significantly attenuated in the inner tissues of a living body, the eyes, the brain, the skin cells, or the myriads of nerve fiber terminals that end up on the outer epidermis, are directly exposed to the field intensities measured externally on the surface of the living tissue.

It has been shown that tissue preparations (such as bovine fibroblasts or chicken tendons) respond to externally applied pulsed or sinusoidal ELF electric fields (by changes in DNA or protein synthesis rates, proliferation rates, alignment with respect to the field direction, etc), at very low thresholds ~10^−3^ V/m^1^,^36^,^37^,^38^. These thresholds are very close to those predicted by the present study.

Except for direct electric field exposure by an external field, there can be an electric field within tissues induced by an externally applied oscillating magnetic one, which as explained penetrates living tissue with zero attenuation. Tuor *et al*.^34^ measured ELF magnetic fields from cell phones on the order of 1 G (=10^−4^ T) at 217 Hz. This can induce electric fields on the order of ~0.1 V/m within the human body, as can be shown by application of Maxwell’s law of electromagnetic induction:


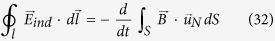


(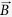, 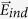, the magnetic and the induced electric field intensities respectively, 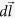 an incremental length along a closed path *l* of induced electric field circulation enclosing a surface S. 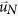 is the unit vector vertical to the surface *S*).

Assuming 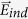 parallel to and independent of *l*, 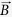 vertical to and independent of *S*, and *l* a circular path of radius *α* including the surface *S*, Eq. 32 becomes:


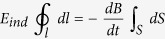


which gives:


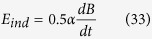


(*E*_*ind*_ in V/m, *B* in T, *α* in m).

By replacing in the last equation *α* = 0.20 m (a reasonably large radius for a circumference within an adult human body), and 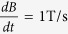, [according to Tuor *et al*.^34^], we get *E*_*ind*_ ~ 0.1 V/m. This is the induced electric field intensity within a human body by the 217 Hz pulses of mobile telephony, and it is about ten times larger than the minimum estimated value able to initiate biological effects at this frequency according to Condition 30.

## Discussion

In the present study we showed that polarized EMFs/EMR, such as every type of man-made EMF, have the ability to create interference effects and amplify their field intensities at specific locations where constructive interference occurs, and that this phenomenon cannot occur with natural EMFs/EMR which are not polarized.

Any location at equal distances from identical sources (antennas), in other words any location along the midperpendicular to the distance *d* between the two sources, is a location of constructive interference and increased field and wave intensities. As the number of sources (e.g. antennas) increases, the amplification of the resultant field intensities (*E*, *B*) at certain locations increases too (Eq. 19), and for a large number of sources field intensities may become very sharp. This explains theoretically the detected “hot spots” from mobile telephony base stations in urban environments^21^. The result of field superposition at those locations are standing waves (i.e. they do not change with time) when the two or more sources of the same polarization are in addition coherent (i.e. same frequency, same phase difference). Within biological tissue, at those locations of constructive interference we can have increased biological activity due to the polarized EMFs.

The most usual case is, when the multiple incident fields/waves are of the same polarization but not coherent (i.e. different frequency and/or varying phase difference), as e.g. the waves from all different radio, television, and mobile telephony antennas vertically oriented. Then, the resultant fields/waves are not standing but timely varying, creating momentary constructive interference at unpredictably different locations each moment. This fact may represent an extraordinary ability of man-made/polarized EMFs to trigger biological effects.

Using the forced-oscillation mechanism^19^,^20^ we showed that the resultant force exerted on the S4 sensors of electrosensitive ion channels on cell membranes by several ions forced to oscillate on parallel planes and in phase by an applied polarized EMF (and even more by constructively superimposed fields from several polarized EMF-sources), is able to irregularly gate these channels. The result can then be the disruption of the cell’s electrochemical balance, leading to a variety of biological/health effects^28^. This is in contrast to the null force exerted by any number of ions oscillating on non-parallel random planes and with different phases from each other due to any number of non-polarized applied EMFs, and in contrast to the null force exerted by the random thermal movement of the same ions^20^,^26^.

In experiments testing the role of different polarization types on the biological activity of RF EMR, exposure of E. coli to 51.76 GHz radiation resulted in inhibition of DNA repair when linear or right-handed circularly polarized radiation was used, while left-handed circularly polarized radiation caused no effects. Exposure to 41.32 GHz similar EMR was reported to reverse the effect: In this case, only linear or left-handed circularly polarized radiation inhibited the DNA repair^39^. In both frequencies, the right-handed or the left-handed circularly polarized radiation induced a greater effect than the linearly polarized radiation. When the structure of the DNA was altered by ethidium bromide intercalation, a change in intensity of the effect of polarization was reported^40^. Chromatin condensation (a sign of cell death) was induced by elliptically polarized 36.65 GHz microwave radiation. The effect increased with intensity. Right-handed polarization induced a stronger effect than left-handed^41^. These experiments show that not only linear but circular and elliptical polarizations are important parameters for the biological action of EMR, and that molecular structure of biomolecules may be important for the interaction between polarized EMF and the biological tissue. In all these studies there was no comparison with unpolarised field of identical other parameters, but only comparison between different types of polarization. Again, it is important to note that circularly and elliptically polarized 50–60 Hz EMFs are formed around 3-phase power transmission lines.

Experiments with non-polarized and polarized EMFs/EMR of identical other characteristics (intensity, frequency, waveform, etc) on certain biological models should be performed to test the validity of the present theoretical study. This should be the subject of a future experimental study.

The present theoretical analysis shows that polarized man-made EMFs/EMR can trigger biological effects while much stronger and of higher energy (frequency) unpolarized EMFs/Non-Ionizing EMR (e.g. heat, or natural light) cannot.

This is the reason why polarized microwave radiation of maximum power 1 W emitted by a mobile phone can damage DNA and cause adverse health effects^2^,^3^,^5^,^6^,^35^, while non-polarized infrared, visible, and ultraviolet radiation from a 100 W light bulb, or ~400 W infrared and visible EMR from a human body^14^,^16^, cannot. Similarly with solar EMR the intensity of which incident on a human body (~8–24 mW/cm^2^) is hundreds of times higher than radiation intensity incident from e.g. a cell phone on a user’s head/body during a usual phone-conversation with the handset in touch with the head (less than 0.2 mW/cm^2^), or incident intensities from other RF, ELF sources of human technology^6^,^7^,^12^,^13^. The total daily duration of human exposure to the sunlight is also much longer normally than the total daily duration of cell phone exposure during conversations^5^,^6^,^12^,^13^. Moreover the frequency (energy) of sunlight is also significantly larger than any man-made RF or ELF frequencies. Yet, there are no adverse biological effects due to normal/non-excessive exposure to sunlight. On the contrary, it is beneficial and vital/necessary for human/animal health, in contrast to cell phone radiation. Similarly, there are no adverse biological effects due to exposure (mainly in the infrared and visible regions) from one human body to another (with an incident intensity ~20 mW/cm^2^)^16^. Although all animals on Earth have adapted throughout evolution to exposures to EMFs from the sun and the earth, these fields are non-polarized (even though natural light may become partially polarized in a small average degree due to atmospheric scattering or reflections). Moreover, terrestrial electric and magnetic fields are mainly static, emitting very weak non-polarized ELF radiation due to slight variations in their intensities. However, larger variations on the order of 20% of their normal intensities due to solar activity with a periodicity of about 11 years result in increase of human/animal health incidents^15^. Therefore, living organisms on Earth are adapted to natural (non-polarized or even partially polarized) EMFs since the beginning of life, but not to variations in their normal intensities on the order of 20%, and thus we would not expect them to adapt to man-made (totally polarized) EMFs/EMR. The present study explained how this difference in polarization results in corresponding differences in biological activity between natural and man-made EMFs.

Increased biological activity does not necessarily result in observable biological/health effects, since there are adaptive mechanisms operating at cellular-tissue-organism levels in response to ever occurring changes. However, these mechanisms may not always be totally effective, especially when the organism is under additional stress or increased metabolic needs (e.g. sickness, childhood/development, old age, etc.). Then exposure to polarized (man-made) EMFs may considerably increase the probability for the initiation of adverse health effects. The effect of polarized EMF-exposure may even be beneficial in certain cases of applied static or pulsed electric or magnetic fields of specified orientation and intensities that enhance the action of endogenous physiological fields within living cells/organisms e.g. during development, wound healing, bone fracture healing etc.^38^,^42^.

The role of polarization in the ability of EMFs/non-ionizing EMR to induce biological effects, as described in the present study, is - up to today - largely underestimated in the EMF-bioeffects literature. Thus, we believe that the present study contributes significantly towards a better understanding of the mechanisms underlying EMF-bioeffects.

## Additional Information

**How to cite this article**: Panagopoulos, D. J. *et al*. Polarization: A Key Difference between Man-made and Natural Electromagnetic Fields, in regard to Biological Activity. *Sci. Rep*. **5**, 14914; doi: 10.1038/srep14914 (2015).
